# Characterization of Type-II Acetylated Cellulose Nanocrystals with Various Degree of Substitution and Its Compatibility in PLA Films

**DOI:** 10.3390/polym9080346

**Published:** 2017-08-06

**Authors:** Feng Dong, Meiling Yan, Chunde Jin, Shujun Li

**Affiliations:** 1 Key Laboratory of Bio-based Material Science and Technology of Ministry of Education, Northeast Forestry University, Harbin150040, China; fengdong0829@gmail.com (F.D.); meilingyan123@gmail.com (M.Y.); 2 Light Industry and Textile School, Qiqihar University, Qiqihar161006, China; 3 Key Laboratory of Wood Science and Technology, Zhejiang A & F University, Hangzhou311300, China; jincd@zafu.edu.cn

**Keywords:** acetylation, cellulose nanocrystals, degree of substitution, polylactic acid, composite film, compatibility

## Abstract

In order to decrease the self-agglomeration and improve the hydrophobic properties of type-II acetylated cellulose nanocrystals (ACNC II), various degree of substitution (DS) values of ACNCs were successfully prepared by a single-step method from microcrystalline cellulose with anhydrous phosphoric acid as the solvent, and acetic anhydride as the acetylation reagent, under different reaction temperatures (20–40 °C). To thoroughly investigate the DS values of ACNC II, analyses were performed using Fourier transform infrared spectroscopy (FT-IR), ^13^C cross polarization-magic angle spinning (CP-MAS) nuclear magnetic resonance (NMR), and X-ray photoelectron spectroscopy (XPS). At a reaction temperature of 40°C, the highest DS value was successfully obtained. XRD proved that the crystal structure of ACNC II with various DS values was maintained after acetylation. TEM showed the threadlike shape for ACNC II with various DS values. The ACNC II with various DS values was introduced into a polylactic acid (PLA) matrix to produce PLA/ACNC composite films, which showed improved rheological and thermal properties. This improvement was primarily attributed to good dispersion of the ACNC II, and the interfacial compatibility between ACNC II and the PLA matrix. This study aims to analyze the compatibility of ACNC II with various DS values in the PLA matrix by microstructure, crystallization, and rheological and thermal tests.

## 1. Introduction

Nowadays, cellulose nanocrystals (CNCs) have attracted considerable attention because of their valuable properties, such as high mechanical properties, stiffness, and surface area [1]. CNCs can be fabricated by several strong or weak acids, and the fabricated CNC have different polymorphs of cellulose (cellulose I and cellulose II) [2]. Generally, cellulose I is natural cellulose from different sources. Cellulose II can be obtained from cellulose I by mercerization or regeneration [3]. Recently, several methods were put forward to prepare CNC II [4,5,6]. Unlike cellulose I, cellulose II has an antiparallel strand arrangement and monoclinic lattice arrangement, so the intermolecular hydrogen bonding is more complicated in cellulose II compared with cellulose I [7]. Apart from the hydrogen bonds, cellulose II has its glucopyranose rings stacked with each other by hydrophobic interactions along the (1-10) plane, thereby resulting in an increased density of hydroxyl groups on the surface, which leads to increased hydrophilicity [8]. The CNC II particles often aggregate easily due to the increased hydrogen bonding [9]. A variety of modification strategies have been proposed to remedy the problems [10]. 

Acetylation, a type of chemical modification often used for this purpose, functions by replacing the hydroxyl groups of cellulose with less hydrophilic acetyl groups [11]. Lin et al. [12] reported that acetylated cellulose nanocrystals (ACNCs) exhibited reduced polarity compared with unmodified CNC. Jonoobi et al. [13] reported that acetylated nanofibers with degree of substitution (DS) values of 1.07 showed more hydrophobic properties compared with non-acetylated nanofibers. Li et al. [14] obtained the ACNC with a DS value of 0.28. Suflet et al. [15] produced the cellulose phosphate with a DS of ≈1. ACNCs show potential application as compatible reinforcement and nanofillers in nanocomposite production [16,17], while acetylation did not affect the nanocomposite properties, as expected, due to the lower DS on cellulose nanocrystals [13]. Up until now, there has been comparatively little research on ACNC II with various DS.

Polylactic acid (PLA), a bio-based and biodegradable polymer produced from renewable resources, is easily processed into a variety of desired shapes. These processes include injection molding, extrusion to obtain films, or spinning into fibers [18]. PLA is a comparatively brittle and stiff polymer with low deformation at break [19]. Its poor thermal and mechanical resistance and limited gas barrier properties are the main limitations for the application of PLA in packaging and biomedical materials [20,21]. In order to overcome its drawbacks, combining PLA with ACNC was developed to modify PLA and improve the combined properties. Many studies of ACNCs as compatible reinforcements in PLA have been carried out [12,13,22,23,24]. Previously published papers mainly focused on the physical, mechanical, rheological and thermal properties of PLA nanocomposites reinforced with ACNC I. To the best of our knowledge, ACNC II obtained by a sing-step method under different reaction temperatures (20–40 °C) has not been investigated at present. Moreover, this kind of ACNC II with a higher DS has not been compounded in PLA-based composites to modify PLA.

In this study, three methods for calculating the DS of ACNC II were adopted, including Fourier transform infrared spectroscopy (FR-IR), X-ray photoelectron spectroscopy (XPS), and solid-state ^13^C cross polarization-magic angle spinning (CP-MAS) nuclear magnetic resonance (NMR). In addition, the microstructure, morphological characterization, and crystallization behavior of ACNC II with various DS values were investigated in detail. Finally, the effect of ACNC II with various DS incorporations on the microstructure, crystallization behavior, rheological and thermal properties of PLA/ACNC composite films was investigated in detail. The good compatibility of the PLA/ACNC composite films is expected to have potential in biomaterials or packaging applications.

## 2. Materials and Methods

### 2.1. Materials and Reagents 

The polylactide (PLA 4032D) was obtained from NatureWorks (Minnetonka, MN, USA). Its specific gravity density is 1.24 g/cm^3^. Microcrystalline cellulose (MCC, product number: 68005761), polyphosphoric acid, phosphoric acid, and acetic anhydride were purchased from Shanghai Sinopharm Chemical Reagent Co., Ltd. (Shanghai, China). The PLA pellets were dried in a vacuum oven at 60 °C for 48 h before use.

### 2.2. Preparation of CNC and ACNC

Acetylated cellulose nanocrystals (ACNCs) were prepared from microcrystalline cellulose (MCC) according to our previous study [25]. The reaction temperature was set between 20–40 °C, because MCC could not homogeneously disperse in the system under a lower temperature and could significantly hydrolyze under higher temperature. First, polyphosphoric acid and 85% phosphoric acid were mixed in a flask for 1.5 h at 48 °C, and then cooled to 3 °C to prepare the anhydrous phosphoric acid system.

The MCC (10 g) and anhydrous phosphoric acid system (90 g) were mixed for 5 min, and 15 mL acetic anhydride was added and stirred in flasks for 3 h at 20 °C, 30 °C, or 40 °C (coded ACNC-20 °C, ACNC-30 °C, and ACNC-40 °C). For comparison, the MCC and anhydrous phosphoric acid system without acetic anhydrous was stirred at 40 °C for 3 h as a control (CNC). Finally, aqueous suspensions of CNC and ACNC were obtained by centrifuging and dialysis. The CNC and ACNC suspensions were lyophilized at −55 °C to obtain white, flaky, and lamellar products. 

### 2.3. Acetylated Extent of ACNC

The extent of acetylation was calculated using three spectroscopic technologies as follows, and compared by the degree of substitution (DS) values of the ACNCs.

#### 2.3.1. Fourier Transform Infrared (FT-IR) Spectroscopy

The organic functional groups of the CNC and ACNC were analyzed with Fourier Transform Infrared Spectroscopy (FT-IR) using a Nicolet Magna 560 spectrometer with an attenuated total reflectance (ATR) accessory. Spectra were recorded between 4000 and 650 cm^−1^ at a resolution of 4 cm^−1^ with 32 scans per sample. The DS of ACNC samples could be calculated through normalization of the absorbance peak area of the ester at 1736 cm^−1^, with the area underneath the absorbance peak of the cellulose repeat unit centered at 1162 cm^−1^ [26]. The normalization proceeds were calculated using the following Equation (1):

(1) $$\frac{a_{1736}}{a_{1162}}=\frac{e\left(1736\right)c_{E}}{e\left(1162\right)c_{R}}=B\frac{c_{E}}{c_{R}}=B\left(N_{E,RU}\right)$$

where a_1736_ and a_1162_ are the areas underneath the peaks at 1736 and 1162 cm^−1^, respectively, and c_E_ and c_R_ are the concentrations of the ester groups and cellulose ring structures, respectively. e(1736) and e(1162) are the molar absorptivities of the peaks corresponding to the ester group at 1736 cm^−1^ and the C–O–C cellulose ring stretch at 1162 cm^−1^. B is a constant, and N_E, RU_ is the number of esters per cellulose repeat unit, i.e., the D.S. values.

#### 2.3.2. ^13^C CP-MAS NMR Spectroscopy

The solid state ^13^C cross polarization-magic angle spinning (CP-MAS) nuclear magnetic resonance (NMR) spectrum of the ACNC was recorded at room temperature on a Bruker DRX-400 spectrometer. The sample was packed in a 4-mm-diameter zirconia rotor. The spectra operated at a frequency of 75.48 MHz under a 7.04 T static field. All spectra were recorded with an MAS rate of 5 kHz, a contact time of 1000 μs, and a repetition time of 5 s. The unmodified CNC exhibited a pattern typical of cellulose, with resonances at C1, C4, C6, and C2/C3/C5 positions, as described in previous literature [27]. After acetylation, the carbonyl of the ester bond (α) and the terminal methyl carbon (β) of the fatty acid chains appeared. The degree of substitution was also calculated as a ratio of the integrals using the following Equation (2):

(2) $$DS_{\alpha /\beta }=E\times \frac{I_{\alpha /\beta }}{I_{2,3,5}}$$

where I_α/β_ is the integral area of the carbonyl of the ester bond or terminal methyl carbons, I_2,3,5_ is the sum of the areas of the carbons of the cellulose (C2/C3/C5), and E is a constant, which could be calculated from the ^13^C CP-MAS NMR spectrum of cellulose acetate.

#### 2.3.3. X-ray Photoelectron Spectrometry (XPS)

X-ray Photoelectron Spectrometry (XPS) of CNC and ACNC samples at different reaction temperatures was conducted using a PHI5700 spectrometer and Al Kσ radiation (hν = 1486.6 eV). Detailed analysis of C1s and O1s of the samples was performed over 279–292 and 525–537 eV, respectively. The binding energy scale shifted to carbon atoms bound to a hydroxyl group in cellulose at 284.6 kV. The C1s spectrum of cellulose was resolved into several components, assigned to C1(C−C/C−H), C2(C−O), C3(O−C−O/C=O), and C4(O−C=O) at about 285.0, 286.5, 288.05, and 289.05kV, respectively [28]. The DS of ACNC was calculated from the peak areas using the following Equation (3):

(3) $$\mathrm{DS}=\frac{O-C=O_{}\mathrm{atomic}\%}{{\mathrm{Cellulose}}^{}{\left(C-O+O-C-O\right)}^{}\%/6}$$

where atomic O–C=O is the number of occurrences by substitution, and Cellulose (C–O + O–C–O) is the amount of cellulose.

### 2.4. X-ray Diffraction (XRD)

The X-ray diffraction (XRD) of CNC and ACNC samples at different reaction temperatures was analyzed between 2θ = 5° and 45° with step increment 2θ = 0.02° in a D/max-250x X-ray Diffractometer (Rigaku Denki, Tokyo, Japan) using a Cu *K*α(40 kV/35 mA).

### 2.5. Microscopy

The morphology, shape, and diameter (calculated on a series of 50 fibers) of ACNC samples were evaluated using a field emission-scanning electron microscope (FE-SEM, Oregon, USA). A drop of ACNC suspension was mounted onto copper, then freeze-dried at -50 °C. The freeze-dried samples were coated with gold and examined under FEI Sirion FE-SEM at an acceleration voltage of 20 KV. The state of dispersion of the ACNC samples was examined using a Hitachi 7560 Transmission electron microscope (TEM, Tokyo, Japan) at 100 KV. A drop of the diluted ACNC suspension was deposited onto carbon-coated grids and allowed to dry at room temperature. The sample was negatively stained with a 2% uranyl acetate aqueous solution for several minutes prior to use.

### 2.6. Redispersion Studies

The redispersibility of the CNC and ACNC samples was observed by preparing dispersions in a series of solvents with decreasing polarity (water > methanol > chloroform > *n*-butanol) at a concentration of 10 mg/mL. Dispersions were sonicated for 10 min.

### 2.7. Preparation of PLA/ACNC Composite Films

The composite films were prepared by solvent casting method. Specific amounts of ACNC, dispersed in CHCl_3_ at room temperature, were added along with 4 g PLA dissolved in 50 mL CHCl_3_ with magnetic stirring. The composite solutions containing ACNC at 0.5 wt% loading at 20, 30, and 40 °C were prepared. The solutions were then cast onto a glass mold and evaporated at ambient temperature for 1 d. The composite films were peeled from the mold, dried in a vacuum desiccator at 60 °C for 2 d, and coded as PLA-20-ACNC, PLA-30-ACNC and PLA-40-ACNC, respectively. The plain PLA film was used as a comparison.

### 2.8. Rheological Characterization of PLA/ACNC Composite Films

A rotational rheometer (AR2000, TA Instruments, New Castle, DE, USA) was used in parallel plate geometry (25 mm diameter) for rheological characterization according to the literature [23], with some modifications. The linear viscoelastic region was determined using strain sweep between the frequency of 0.1 and 100 rad/s at 180 °C. The results included complex viscosity (η*), storage modulus (G′), and loss modulus (G′′) as key parameters characterizing the viscoelastic behavior of the PLA/ACNC composite films. 

### 2.9. Thermal Measurements of PLA/ACNC Composite Films

Thermogravimetric analysis (TGA) was performed using a Netzsch TA4 (Selb, Germany) thermal analyzer from 20–500 °C at a rate of 10 °C /min. All composite films were dried in a vacuum desiccator at 60 °C before testing. The differential scanning calorimeter (DSC) of composite films were measured on a Netzsch 204E (Selb, Germany) at a heating rate of 10 K/min and a range of 0–200 °C in a nitrogen atmosphere [29]. The glass transition temperature (Tg), melting temperature (Tm), enthalpies of fusion (ΔHf), and crystallization (ΔHc) of the different films were measured from the first and second heating, and the degree of crystallinity (Xc) was evaluated using the following Equation (4):

(4) $$X_{c}=100\times \left[\frac{\left(\Delta H_{m}-\Delta H_{c}\right)}{\Delta H_{f}}\right]$$

Where ΔH_f_ is the enthalpy of fusion; ΔH_c_ is the enthalpy of crystallization; and ΔH_m_ is the enthalpy of fusion of a wholly crystalline PLA (93.6 J/g) [30].

### 2.10. Scanning Electron Microscope (SEM) of PLA/ACNC Composite Films

The samples were prepared by dropping a 5 mm × 5 mm piece cut from the center of the film into liquid nitrogen and allowing the piece to equilibrate under the liquid nitrogen. The film piece was fractured into several smaller pieces with a pre-chilled razor blade held in a vice grip. The cross-sections of the samples, coated with gold, were observed using SEM.

### 2.11. X-ray Diffraction (XRD) of PLA/ACNC Composite Films

The crystal structure of the PLA/ACNC composite films was analyzed using X-ray diffraction (XRD). Measurements were obtained on a D8 ADVANCE XRD (Karlsruhe, Germany) in the range of 5° to 70°, with a step size of 0.02° at a scan speed of 1 s^−1^.

## 3. Results

### 3.1. Spectrograms of ACNCs 

**Figure 1:**
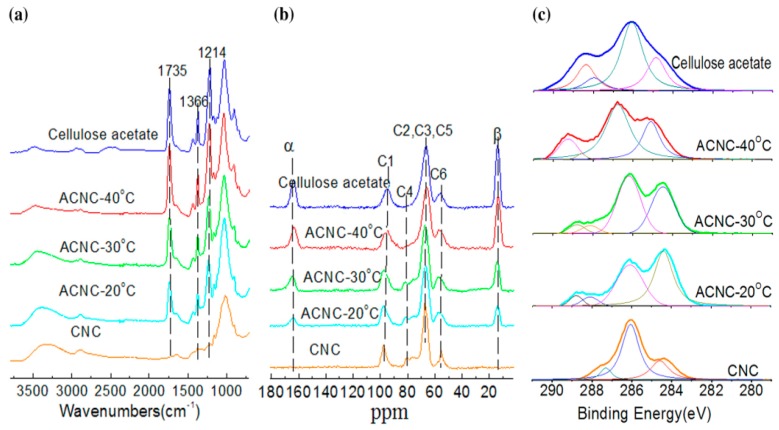
Spectrograms of cellulose acetate for acetylated cellulose nanocrystals (ACNCs) at the following temperatures: ACNC-40 °C, ACNC-30 °C, ACNC-20 °C, and cellulose nanocrystals (CNC): (**a**) Fourier transform infrared spectroscopy (FT-IR) spectra. (**b**) ^13^C cross polarization-magic angle spinning (CP-MAS) nuclear magnetic resonance (NMR) spectra. (**c**) X-ray photoelectron spectroscopy (XPS) spectra.

Figure 1a shows the FT-IR spectra of the unmodified CNC and ACNC samples. For the unmodified CNC, the absorption bands at 3364 and 2897 cm^−1^ are attributed to cellulose vibration. After acetylation, all the ACNC spectra show favorable results, with ester group vibrations appearing at 1735 (C=O), 1366 (CH_3_), and 1214 cm^−1^ (C–O) [13]. The absorption peak at 3332cm^−1^ (–OH) decreased as the reaction temperature increased. When the reaction temperature was between 20–40 °C, acetylation reaction occurred between the hydroxyl group and the acetic anhydride. Thus, the peak of hydroxyl groups was significantly reduced. The sample ACNC-40 °C exhibited much more pronounced ester peaks in the FT-IR spectra. The extent of the esterification reaction is accompanied by the appearance of new absorption peaks at 1735, 1366, and 1214 cm^−1^, referred to as C=O, CH_3_, and C–O, respectively. Figure 1b shows the ^13^C CP-MAS NMR spectra obtained from freeze-dried CNC and ACNC. Similar to previous literature [31], the CNC spectrum exhibited typical cellulose signals at C1 (98 ppm), C4 (80 ppm), C6 (57 ppm), and C2/C3/C5 (66 ppm). After acetylation, the carbons of the acetyl groups were clearly identifiable at 165 and 14 ppm, which corresponded to α and β, and confirmed the successful acetylation of ACNC. XPS measurements were gathered to detail the surface compositions derived only from the superficial layer of CNC, ACNC, and cellulose acetate samples. As shown in Figure 1c, C1s spectra were resolved into several components, categorized as C−C/C−H (285 eV, where the binding energy scale was changed according to this value as the starting position,) C−O (286.5 eV), O−C−O/C=O (288.05 eV), or O−C=O (289.0 eV) [32]. 

### 3.2. Degree of Substitution (DS) of ACNCs

According to the Figure 1a, the N_E, RU_ of ACNC-20 °C, ACNC-30 °C and ACNC-40 °C was 0.59, 1.06 and 1.56, respectively, the N_E, RU_ of cellulose acetate was 1.66 (given in chemical composition). The DS values of the ACNCs were 0.63, 1.14 and 1.64, respectively, calculated using Equation (1) under different acetylation temperatures. Variations in DS within N_E, RU_ reached a maximum at 40 °C. According to the Figure 1b, the DS_α_ of ACNC-20 °C, ACNC-30 °C and ACNC-40 °C was 0.51, 1.12 and 1.36, respectively; while the DS_β_ of ACNC-20 °C, ACNC-30 °C and ACNC-40 °C was 0.55, 0.90 and 1.40, respectively. It was clearly observed that the DS values increased as the reaction temperature increased. The DS values of ACNCs were calculated using Equation (2) under different acetylation temperatures. The DS of ACNC-40 °C was about 1.40. The higher the DS, the fewer hydroxyl groups remained in cellulose samples. According to Figure 1c, the DS of ACNC-20 °C, ACNC-30 °C and ACNC-40 °C was 0.56, 0.65 and 1.21, as calculated using Equation (3) under different acetylation temperatures. These results were consistent with FT-IR and ^13^C CP-MAS NMR spectra, where a higher reaction temperature contributed to acetylated reaction. The calculated DS values of ACNCs were listed in Table 1; as shown, a higher reaction temperature contributed to an acetylated reaction.

### 3.3. X-ray Diffraction Analysis of CNC and ACNCs

**Figure 2:**
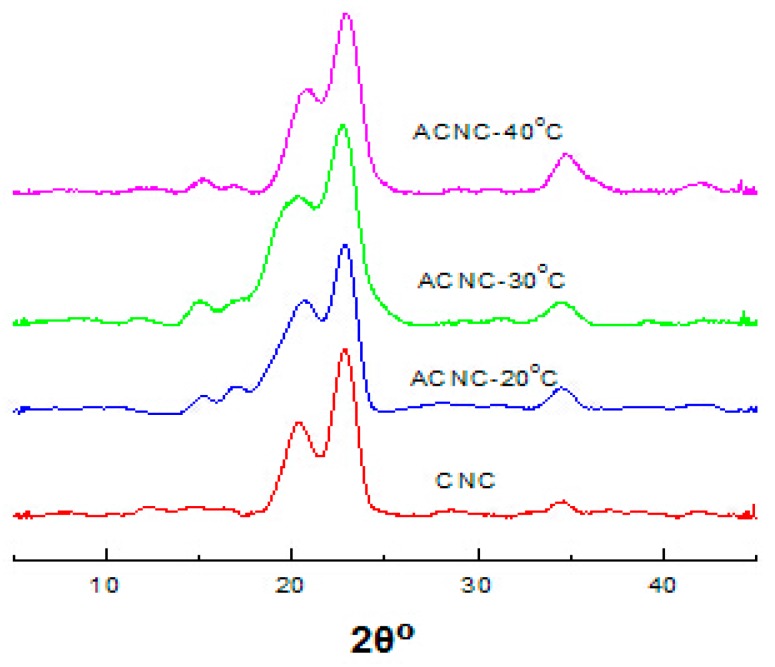
X-ray diffraction (XRD) patterns of ACNC-40 °C, ACNC-30 °C, ACNC-20 °C, and CNC.

XRD patterns for CNC, ACNC-20 °C, ACNC-30 °C and ACNC-40 °C are shown in Figure 2. It can be seen that there were high peaks appearing at about 12.4°, 20.5° and 22.2°, in which 12.4° and 20.5° were due to the 101 side, and 22.2° was due to the 002 side of cellulose [25]. All of the characteristic peaks demonstrate that both the CNC and ACNCs had the typical type II cellulose, and the acetylation did not change the crystal structure. Moreover, with the increased DS values of ACNCs, the XRD patterns were still rather similar to that of the CNC, signifying that the crystal structure was unchanged.

### 3.4. Microscopy Analysis of ACNCs

**Figure 3:**
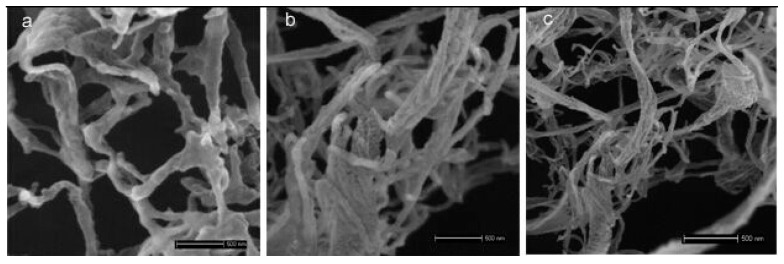
FE-SEM images: (**a**) ACNC-20 °C, (**b**) ACNC-30 °C, (**c**) ACNC-40 °C.

SEM images of the freeze-drying ACNCs are shown in Figure 3. From the image, it is evident that the threadlike morphology and crystal structure were preserved. Single cellulose nanofibrils of ANC-20 °C, ANC-30 °C and ANC-40 °C were observed with diameters of 33.94 ± 16, 32.98 ± 9 and 24.67 ± 7nm, respectively. It seemed the filamentary shape was due to the tendency of ACNC to agglomerate and form strong hydrogen bonds as the water sublimates during the freeze-drying process [33]. It can be seen that the diameter of the ACNCs decreased with increased DS values of ACNCs, which probably resulted from the partial solubility of the cellulose molecules during esterification [34].

**Figure 4:**
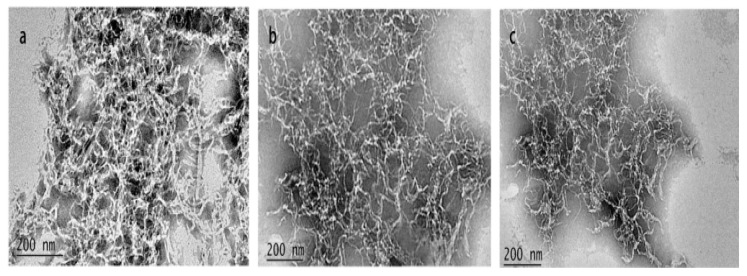
TEM images: (**a**) ACNC-20 °C, (**b**) ACNC-30 °C, (**c**) ACNC-40 °C.

TEM images of ACNCs are shown in Figure 4. It can be seen that the obtained ACNCs each had a threadlike shape. After the acetylation, the diameters of the ACNC particles were still between 20–30 nm. Also, the length distribution of ACNC-40 °C was more uniform than that of ACNC-20 °C or ACNC-30 °C.

### 3.5. Redispersibility of CNC and ACNCs

**Figure 5:**
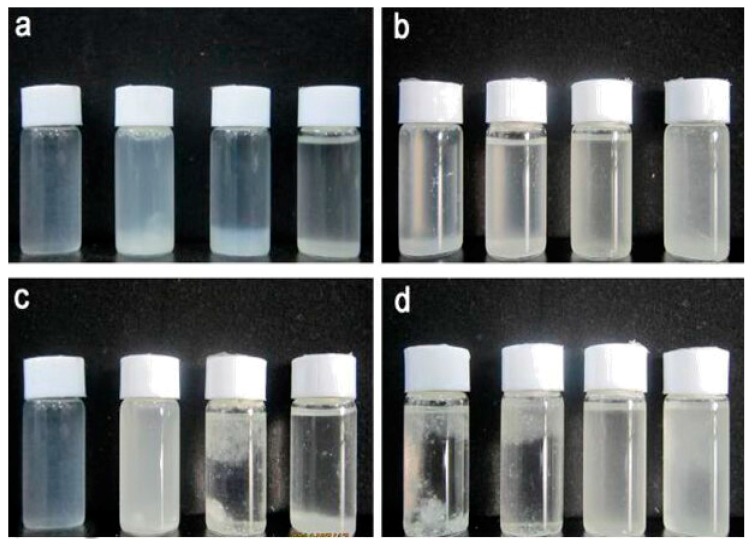
Photographs of CNC or ACNCs after redispersion: (**a**) CNC was dispersed in water, methanol, chloroform, and n-butanol. (**b**) ACNC-40 °C was dispersed in water, methanol, chloroform, and n-butanol. (**c**) CNC, ACNC-20 °C, ACNC-30 °C, and ACNC-40 °C were dispersed in water. (**d**) CNC, ACNC-20 °C, ACNC-30 °C, and ACNC-40 °C were dispersed in chloroform.

In order to better observe the redispersibility of CNC and ACNCs, photographs of the different suspensions are summarized in Figure 5. As shown in Figure 5a and b, with increased DS values of ACNCs, the dispersion of CNC or ACNCs in water decreased; however, in chloroform, they increased. The CNC was not able to disperse within chloroform and n-butanol (Figure 5c). The modified sample (Figure 5d) did migrate into the chloroform, suggesting that ACNC-40 °C was successfully modified, as also indicated by the FT-IR results. An attractive property of ACNCs is their ability to form stable colloidal suspensions or liquid crystals in appropriate solvents [35]. 

### 3.6. Rheological Properties of PLA/ACNC Composite Films

**Figure 6:**
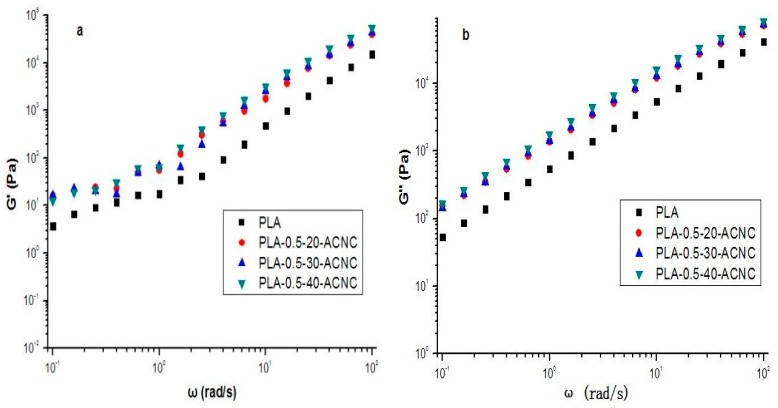
Storage (*G*′) and loss (*G*′′) modulus of polylactic acid (PLA)/ACNC composite films: (**a**) Storage modulus. (**b**) Loss modulus.

The storage modulus (*G*′), loss modulus (*G*′′), and complex viscosity (η*) of PLA/ACNC composite films are presented in Figure 6a and Figure 6b. The *G*′ and *G*′′ of PLA/ACNC composite films all increased with increasing frequency as compared to the plain PLA. It is evident that the acetylation did improve the *G*′ and *G*′′ of the PLA matrix; particularly, the PLA incorporation of ACNC-40 °C presented the highest *G*′ and *G*′′. This phenomenon might be possibly explained by analyzing their microstructure. The hydrophobicity of ACNCs improves as the DS values of ACNCs increased, which restricted agglomeration between ACNC and PLA, and resulted in a better dispersion and compatibility in the PLA matrix. Therefore, the improvement increases the storage modulus of PLA films [36].

**Figure 7:**
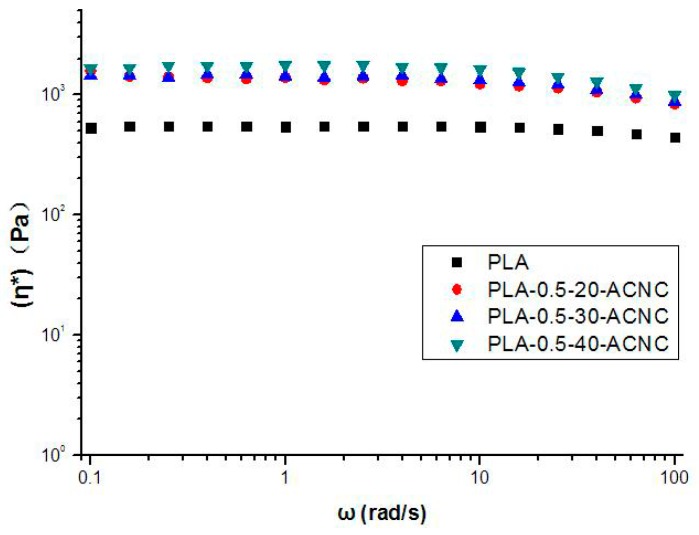
Complex viscosity (η*) of PLA/ACNC composite films as a function of frequency.

From Figure 7, it is observed that complex viscosity is higher for all the PLA/ACNC composite films than plain PLA. The complex viscosity increased as the DS values of ACNCs increased. This was probably due to the increase of dispersion and compatibility between ACNC and PLA causing consolidation in the polymer chain network [36,37]. This consolidated effect is more pronounced when ACNC-40 °C is used in PLA films, which confirms better interfacial compatibility. 

### 3.7. Thermal Properties of PLA/ACNC Composite Films 

**Figure 8:**
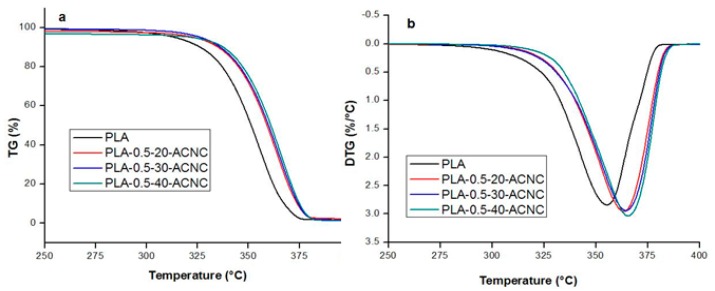
Thermograms of the PLA composite films: (**a**) thermogravimetric analysis curves, (**b**) derivative thermogravimetric curves.

Weight loss (TG) and derivative curves (DTG) of PLA/ACNC composite films are detailed in Figure 8. Plain PLA shows a degradation peak that starts around 314 °C and reaches its maximum at 351 °C. A shift toward higher temperature for the degradation peak was observed due to the presence of ACNC. As the DS values of ACNCs increased, the degradation peaks of the films also significantly increased. When ACNC-40 °C was added to PLA film, the value of the degradation peak was maximized (373 °C). Results showed that the use of higher-DS ACNC improved the thermostability of the PLA film, which is likely due to the increased dispersion of the higher-DS ACNC. The increased dispersion is believed to result from an increased compatibility between ACNC II and the PLA matrix [10].

**Figure 9:**
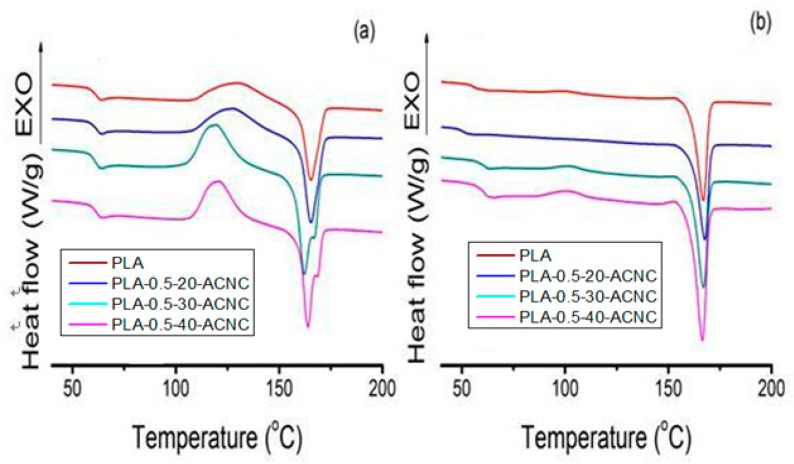
Differential scanning calorimeter (DSC) thermograms of PLA/ACNC composite films. (**a**) first heating scan. (**b**) second heating scan.

DSC first and second heating curves of plain PLA and PLA/ACNC composite films were collected, as shown in Figure 9. Data for glass transition (*T*_g_), melting temperature (*T*_m_), heat of fusion (Δ*H*_m_) and the degree of crystallinity (*X*_c_) are summarized in Table 2. The plain PLA showed a *T*_g_ value of 61.36 °C, and the introduction of ACNC-20 °C, ACNC-30 °C and ACNC-40 °C at 0.5% loading resulted in a slight shift of the *T*_g_ value down to 61.48, 61.65 and 61.79 °C, respectively. The *T*_g_ values suggested that the ACNCs did not affect the translational and rotational backbone motions of the PLA chains and decreased the free volume of amorphous regions [38]. The *T*_m_ value of plain PLA was observed at 160.71 °C, which slightly increased to 161.54 °C when the ACNC-40 °C was introduced into the PLA. This increase may be attributed to the increasing compatibility and formation of cross-linked structures, which act as nucleating sites for crystal growth [39]. The *X*_c_ values of PLA films that had incorporated ACNC-40 °C increased 68.7% compared with the plain PLA. The PLA crystallites increased significantly with the addition of higher-DS ACNCs. The occurrence of such a phenomenon is probably due to increasing numbers of cross-linked structures between ACNC II and PLA, which restricted the amorphous polymer chain mobility and led to better interfacial compatibility [24].

### 3.8. Morphology of PLA/ACNC Composite Films 

**Figure 10:**
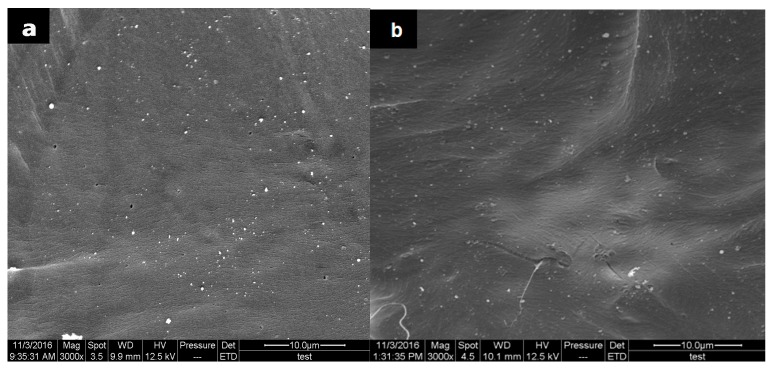
Scanning electron microscope (SEM) cross-sections of PLA/ACNC composite films: (**a**) PLA-0.5-20-ACNC, (**b**) PLA-0.5-40-ACNC.

Figure 10 represented the SEM images of the fracture surfaces of the PLA composite films reinforced with ACNC-20 °C and ACNC-40 °C at 0.5% loading, labeling PLA-0.5-20-ACNC and PLA-0.5-40-ACNC, respectively. Figure 10a showed the fracture morphology of ACNC-20 °C dispersed in the PLA matrix, which exhibited slightly coarse surfaces, but still good miscibility with the matrix. Figure 10b exhibited a better miscibility and interfacial compatibility with the PLA matrix incorporation of the higher-DS ACNCs.

### 3.9. Crystallization of PLA/ACNC Composite Films

**Figure 11:**
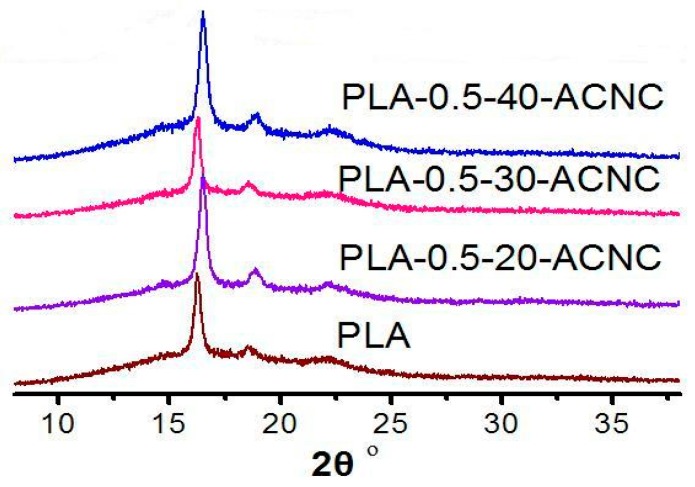
XRD patterns of PLA composite films.

Figure 11 showed the XRD patterns of PLA/ACNC composite films with three main peaks at 2θ = 16.3°, 18.5°, and 22.6°, which correspond to (110/200), (203), and (015) of the homopolymer crystals, respectively. The three peak positions are in accordance with previously reported PLA values [23]. The incorporation of the ACNCs in PLA led to an increase in the intensity of the diffraction peak at ~16.4°, which suggested enhancement of the crystallinity of the PLA. This is probably due to the formation of cross-linked structures between ACNC and PLA. The presence of such interaction probably leads to an ordered arrangement, which subsequently enhances the crystallinity of PLA/ACNC composite films. The change of the intensity of peak at ~22.6° was not significant, and the peak seemed a little sharper compared to the plain PLA, which was probably because of the formation of a new crystalline form by incorporation of ACNCs into the PLA matrix. On the whole, the XRD patterns were still rather similar to that of the plain PLA, signifying that the crystalline structure of the PLA was unchanged. This observation is similar to the results observed by Mukherjee et al. [23], who studied the crystallinity of MCC modified by acetyl chloride in the PLA matrix. 

## 4. Conclusions

Type-II ACNC with various DS values were prepared by a single-step method under different temperatures. DS values were examined by FT-IR, ^13^ C CP-MAS NMR, and XPS. When temperature increased, the DS values of ACNCs increased. The optimal reaction temperature for the highest DS was identified at 40 °C. The PLA films with 0.5 wt % ACNC-40 °C proved to have the most dispersion and compatibility compared with the plain PLA. The results of this study showed that surface acetylation treatment improves the compatibility between ACNC II and PLA. The addition of ACNC-40 °C improved the interfacial compatibility in the observations in rheological tests. The morphology studies showed that relatively good dispersion was achieved, and no agglomerations of ACNCs were observed in the fracture surfaces of PLA/ACNC composite films. The compatibility caused a slight increase in *T*_g_ and *T*_m_, and the DSC results confirmed that ACNCs act as nucleating agents and improve thermostability. Additionally, the crystal structure of the PLA/ACNC composite films was elevated due to the nucleation of ACNCs, but acetylation did not affect the crystalline structure of the PLA matrix in essence.

## Figures and Tables

**Table 1:** Degree of substitution (DS) calculated results of ACNC samples.

Calculation	ACNC-20 °C	ACNC-30 °C	ACNC-40 °C
FT-IR	0.63	1.14	1.64
^13^C CP-MAS NMR(DS_α_)	0.51	1.12	1.36
^13^C CP-MAS NMR(DS_β_)	0.55	0.90	1.40
XPS	0.56	0.65	1.21

**Table 2:** Thermal data for the PLA composite films calculated from the DSC second heating scan.

Sample	*X*_c_ (%)	*T*_g_ (°C)	*T*_m_ (°C)	Δ*H*_m_ (J/g)
PLA	25.26 ± 0.25 ^a^	61.36 ± 0.27 ^a^	160.71 ± 0.55 ^a^	23.49 ± 0.62 ^a^
PLA-0.5-20-ANC	32.52 ± 0.44 ^b^	61.48 ± 0.35 ^b^	160.99 ± 0.61 ^a^	30.24 ± 0.58 ^b^
PLA-0.5-30-ANC	36.37 ± 0.38 ^b^	61.65 ± 0.43 ^b^	162.56 ± 0.65 ^a^	33.82 ± 0.61 ^b^
PLA-0.5-40-ANC	42.62 ± 0.45 ^c^	61.79 ± 0.32 ^b^	161.54 ± 0.57 ^b^	39.64 ± 0.76 ^c^

^a−c^ Different letters in the same column correspond to statistically different samples for a 95% confidence level.
